# Mantle cell lymphoma-like lymphomas in c-*myc*-3'RR/p53^+/−^ mice and c-*myc*-3'RR/Cdk4^R24C^ mice: differential oncogenic mechanisms but similar cellular origin

**DOI:** 10.18632/oncotarget.474

**Published:** 2012-05-15

**Authors:** Pauline Rouaud, Rémi Fiancette, Christelle Vincent-Fabert, Virginie Magnone, Michel Cogné, Pierre Dubus, Yves Denizot

**Affiliations:** ^1^ UMR CNRS 7276, Faculté de Médecine, Limoges, France; ^2^ CNRS and University of Nice Sophia Antipolis, Institut de Pharmacologie Moléculaire et Cellulaire, UMR 6097, Sophia Antipolis, France; ^3^ EA2406, Université de Bordeaux, Bordeaux, France

**Keywords:** Mantle cell lymphoma, c-myc

## Abstract

Mantle cell lymphoma (MCL) is a malignant lymphoproliferative B-cell disorder that does not occur spontaneously in mice but experimental mice model have been developed. Recently two different mice models prone to develop MCL-like lymphomas were generated: c-*myc*-3'RR/Cdk4^R24C^ mice and c-*myc*-3'RR/p53^+/−^ mice. Comparison of their gene expression profiles does not highlight specific differences other than those in relation with their specific mutational status (i.e., Cdk4^R24C^ mutation or p53 mutation). We propose that similarly to typical human MCL and its blastoid or cyclin-D1 variants that correspond to the same genetic entity, MCL-like lymphomas of c-*myc*-3'RR/p53^+/−^ mice and c-*myc*-3'RR/Cdk4^R24C^ mice represent a spectrum of the same entity.

## INTRODUCTION

Mantle cell lymphoma (MCL) is a malignant lymphoproliferative B-cell disorder derived from naïve pregerminal center CD5^+^ cells [1]. MCL is strongly associated with the t(11,14) chromosomal translocation leading to overexpression of cyclin D1. Cyclin D1-negative MCL do no express high amounts of cyclin D1 but elevated cyclin D2 or cyclin D3 ones and share the same secondary genetic aberrations that typical MCL supporting the concept that they correspond to the same genetic entity [1]. MCL does not occur spontaneously in mice but experimental mice model have been developed. Old Eμ-cyclin D1 transgenic mice developed CD19^+^IgM^+^CD5^+^CD23^−^ MCL under stimulation by pristine, a pro-inflammatory tumor promoter [2]. Interleukin 14 alpha (IL-14α)/c-*myc* double transgenic mice developed lymphomas reproducing many features of blastoid variant of MCL [3]. Recently we generated two different mice models prone to develop MCL-like lymphomas. The first one uses the Cdk4-Arg24Cys (R24C) mutation that abolishes the ability of all four INK4 members to bind Cdk4. Disruption of Cdk4 regulation by INK4 while c-*myc* is overexpressed in B-cells (in a c-*myc*-3'RR transgenic background prone to develop Burkitt lymphoma (BL)-like lymphomas [4]) leads to the development (in double mutant c-*myc*-3'RR/Cdk4^R24C^ mice) of lymphoid malignancies closely resembling human MCL [5]. The second is relevant to the frequent loss of p53 function observed in human lymphomas, underscoring its critical role in suppressing the emergence of incipient tumors. Double mutant c-*myc*-3'RR/p53^+/−^ mice developed a wide pattern of lymphomas including MCL-like lymphomas [6]. It remains unclear what are the common molecular and genetic pathways explaining the convergence of these two mice models towards the same lymphoma phenotype. In both situations, MCL-like lymphomas express similar membrane B-cell differentiation markers (B220^+^CD19^+^IgM^+^IgD^+^ CD5^+^CD23^−^) but arise with different kinetics (3 months *vs* 6 months for c-*myc*-3'RR/p53^+/−^ mice and c-*myc*-3'RR/Cdk4^R24C^ mice, respectively), with a different proteomic signature (Cdk6/cyclin D complexes *vs* Cdk4/cyclin D complexes for c-*myc*-3'RR/p53^+/−^ mice and c-*myc*-3'RR/Cdk4^R24C^ mice, respectively), and in relation with a different mutational status (p53 deficiency *vs* disruption of Cdk4 regulation by INK4). Gene arrays have proven effective in establishing molecularly defined subgroups within defined tumor entities. We explored the potential similar biological entity of these two mice models of MCL-like lymphomas by comparing their gene expression profiles.

## MATERIAL AND METHODS

Our research has been approved by the ethics committee review board of our University (Limoges, France) and hospital (CHU Dupuytren, Limoges, France). Animal works has been conducted according to French laws. c-*myc*-3'RR transgenic mice are prone to BL-like lymphomas [4]. In c-*myc*-3'RR mice, c-*myc* is specifically expressed from the pre-B to the mature B-cell stages; the 3'RR being active in late B cell lymphopoiesis [7-9]. Generation of c-*myc*-3'RR/Cdk4^R24C^ and c-*myc*-3'RR/p53^+/−^ mice in similar genetic background and their MCL-like lymphoma development have been previously reported [5, 6]. mRNA was extracted from MCL-like lymphoma cases by sorting tumoral cells with CD19-coupled beads (Miltenyi Biotech, Bergisch Gladbach, Germany). Microarray experiments were done in “Nice - Sophia Antipolis Microarray Facility” (France). Statistical analysis was made with the Bioconductor open source software, particularly its Limma package. The microarray data presented in this article have been submitted to the Gene Expression Omnibus database (www.ncbi.nlm.nih.gov/geo/) under the accession numbers GSE36808.

## RESULTS AND DISCUSSION

The gene expression profile of MCL-like lymphomas of c-*myc*-3'RR/Cdk4^R24C^ mice was compared with the one of c-*myc*-3'RR/p53^+/−^ mice using an array of 44,000 genes. A Log score >2, a Log ratio >2 or <-2 and a p<0.05 was used as cut of. Among these 44,000 genes, only 176 significantly differed (Tables 1, 2, 3 and 4). Noticeably, differential expression of multiple genes involved in growth, metabolism and signalling (97/176, 55.1%, Table 1), diabetes and obesity (31/176, 17.6%, Table 2) and cellular architecture (23/176, 13.0%, Table 3) were found. Thirty two unknown genes significantly differed (32/176, 18.1%, Table 4). Of note some genes can be found both in Table 1, 2 and/or 3.

**Table 1:** Genes implicated in growth, metabolism and signalling processes

			Log2 ratio	p-value	log score
Name	Systematic Name	Description	A-B	A-B	A-B
Tppp	NM_182839	polymerization promoting protein	−5.97	0.03	4.48
Prkcc	NM_011102	protein kinase C, gamma	−4.35	0.03	4.28
Gchfr	NM_177157	GTP cyclohydrolase I feedback regulator	−3.91	0.03	4.14
Chit1	NM_027979	chitinase 1 (chitotriosidase)	−5.14	0.03	4.11
Ahnak	NM_001039959	nucleoprotein (desmoyokin), TV 3	−4.48	0.03	3.95
Trf	NM_133977	transferrin	−4.41	0.03	3.94
Apoc1	NM_007469	apolipoprotein C-I, TV 1	−3.74	0.03	3.94
Macrod2	NM_028387	MACRO domain containing 2, TV 2	2.89	0.03	3.56
Rfx2	NM_009056	regulatory factor X, TV 2	−3.31	0.03	3.52
Ctse	NM_007799	cathepsin E	−4.17	0.03	3.46
Cdkn2a	NM_009877	cyclin-dependent kinase inhibitor 2A, TV 1	4.17	0.03	3.45
Slc40a1	NM_016917	solute carrier family 40, M 1	−5.21	0.03	3.43
Igf1	NM_010512	insulin-like growth factor 1, TV 1	−3.1	0.03	3.43
Igf2bp3	NM_023670	insulin-like growth factor 2 mRNA binding protein 3	2.85	0.03	3.38
Slc2a6	NM_172659	solute carrier family 2, M 6	2.51	0.03	3.31
Ngfr	NM_033217	nerve growth factor receptor	−3.13	0.03	3.28
Camk4	NM_009793	calcium/calmodulin-dependent protein kinase IV	3.42	0.03	3.25
Rtn4rl1	NM_177708	reticulon 4 receptor-like 1	−4.58	0.03	3.19
Ear11	NM_053113	eosinophil-associated, ribonuclease A family, M 11	−2.64	0.03	3.14
Ccnb1	NM_172301	cyclin B1	2.9	0.03	3.11
Rasal1	NM_013832	RAS protein activator like 1 (GAP1 like)	2.67	0.03	3.10
As3mt	NM_020577	arsenic (+3 oxidation state) methyltransferase	−2.71	0.03	3.08
Ppl	NM_008909	periplakin	−2.79	0.03	3.06
Spic	NM_011461	spi-C transcription factor	−3.85	0.03	2.97
Eif2c2	NM_153178	eukaryotic translation initiation factor 2C	3.42	0.03	2.95
Ak4	NM_001177602	adenylate kinase 4, TV 1	3.54	0.03	2.93
Timeless	NM_001164081	timeless homolog (Drosophila),TV 4	2.57	0.03	2.89
Rgs12	NM_173402	regulator of G-protein signaling 12, TV 1	3.1	0.03	2.86
Ccr3	NM_009914	chemokine (C-C motif) receptor 3	−2.74	0.03	2.79
Mrap	NM_029844	melanocortin 2 receptor accessory protein	−2.36	0.03	2.79
Cdc20	NM_023223	cell division cycle 20 homolog	2.26	0.03	2.77
Adarb1	NM_001024837	adenosine deaminase B1, TV 2	2.25	0.03	2.75
Cotl1	NM_028071	coactosin-like 1 (Dictyostelium)	−2.13	0.03	2.69
Trip13	NM_027182	thyroid hormone receptor interactor 13	2.3	0.03	2.65
Atp6v1c2	NM_133699	ATPase, lysosomal V1 subunit C2, TV 2	−2.83	0.03	2.58
Slc43a3	NM_021398	solute carrier family 43, M 3	3.27	0.03	2.46
Sbk1	NM_145587	SH3-binding kinase 1	−2.45	0.03	2.38
Vcam1	NM_011693	vascular cell adhesion molecule 1	−4.91	0.03	2.37
Kcnj16	NM_010604	potassium inwardly-rectifying channel, subfamily J, M 16	−2	0.03	2.33
Lmo1	NM_057173	LIM domain only 1	2.01	0.03	2.30
Osbpl5	NM_024289	oxysterol binding protein-like 5, TV 1	−2.17	0.03	2.30
Tmco6	NM_028036	transmembrane and coiled-coil domains 6	−2.01	0.03	2.30
Serpinb3c	NM_201363	serine (or cysteine) peptidase inhibitor, clade B, M 3C	2.63	0.03	2.25
Trim11	NM_053168	tripartite motif-containing 11	−2.06	0.03	2.24
Slc16a9	NM_025807	solute carrier family 16, M 9	−2.07	0.03	2.23
Fxyd6	NM_022004	FXYD domain-containing ion transport regulator 6	2.13	0.03	2.22
Gstm5	NM_010360	glutathione S-transferase, mu 5	2.52	0.03	2.19
Slc34a1	NM_011392	solute carrier family 34 (sodium phosphate), M 1	−2.82	0.03	2.14
Rnf157	ENSMUST00000149682	ring finger protein 157	2.52	0.03	2.124
Nek2	NM_010892	NIMA (never in mitosis gene a)-related expressed kinase 2	2.57	0.03	2.11
C6	NM_016704	complement component 6	−2.18	0.03	2.05
H60a	NM_010400	histocompatibility 60a	−6.18	0.03	2.04
septin3	NM_011889	septin 3	−6.18	0.03	2.04
Ear10	NM_053112	eosinophil-associated, ribonuclease A family, M 10	−2.2	0.03	1.99
Kctd17	NM_001081367	potassium channel tetramerisation domain containing 17	3.64	0.03	1.91
Casc4	NM_001205369	cancer susceptibility candidate 4, TV 3	2.44	0.03	1.89
Tcp11l2	NM_146008	t-complex 11 like 2	−2.88	0.03	1.83
Mybl2	NM_008652	myeloblastosis oncogene-like 2	2.16	0.03	1.83
Prkar2a	NM_008924	protein kinase, cAMP dependent regulatory, type II alpha	2.1	0.03	1.81
Tcp11	NM_013687	t-complex protein 11, TV 1	−2.04	0.03	1.81
Trerf1	NM_172622	transcriptional regulating factor 1,TV 2	3.07	0.03	1.80
Pvrl2	NM_008990	poliovirus receptor-related 2, TV 1	−4.17	0.03	1.78
Prkar2b	NM_011158	protein kinase, cAMP dependent regulatory, type II beta	−3.46	0.03	1.78
Melk	NM_010790	maternal embryonic leucine zipper kinase	2.23	0.03	1.75
Cdca3	NM_013538	cell division cycle associated 3	2.21	0.03	1.74
B3gnt8	NM_146184	betaGal beta-1,3-N-acetylglucosaminyltransferase 8, TV 1	−2.19	0.03	1.71
Nradd	NM_026012	neurotrophin receptor associated death domain	2.29	0.03	1.68
S100a5	NM_011312	S100 calcium binding protein A5	−2.38	0.03	1.68
Antxr1	NM_054041	anthrax toxin receptor 1	2.25	0.04	1.64
Pear1	NM_028460	platelet endothelial aggregation receptor 1, TV 1	−2.6	0.04	1.62
Akap13	ENSMUST00000136989	A kinase (PRKA) anchor protein 13	−2.23	0.04	1.62
Ebf3	NM_010096	early B-cell factor 3, TV 3	2.86	0.04	1.60
Tk1	NM_009387	thymidine kinase 1	2.46	0.04	1.57
Rgs11	NM_001081069	regulator of G-protein signaling 11	−2.22	0.04	1.57
Gm4910	XM_141816	predicted pseudogene 4910	−2.22	0.04	1.56
Ell3	NM_145973	elongation factor RNA polymerase II-like 3	−3.12	0.04	1.55
Etl4	NM_001081006	enhancer trap locus 4, transcript variant c	3.4	0.04	1.50
Syce2	NM_027954	synaptonemal complex central element protein 2, TV 2	3.17	0.04	1.47
LOC100042049	NR_004442	ribosomal protein L22 like 1 pseudogene	−2.17	0.04	1.38
Sorcs2	NM_030889	sortilin-related VPS10 domain containing receptor 2	−2.12	0.04	1.38
Crip2	NM_024223	cysteine rich protein 2	−4.51	0.04	1.37
Wnk2	NM_029361	WNK lysine deficient protein kinase 2	3.76	0.04	1.36
Samd9l	NM_010156	sterile alpha motif domain containing 9-like	−2.09	0.04	1.36
Hoxb6	NM_008269	homeobox B6	−2.23	0.04	1.35
Rpl22l1	NM_026517	ribosomal protein L22 like 1	−2.02	0.04	1.34
Crip1	NM_007763	cysteine-rich protein 1	−4.05	0.04	1.31
Enkur	NM_027728	enkurin	−3.01	0.04	1.30
Ear2	NM_007895	eosinophil-associated, ribonuclease A family,M 2	−3.45	0.04	1.29
Cdca5	NM_026410	cell division cycle associated 5	2.04	0.04	1.23
Mrc1	NM_008625	mannose receptor, C type 1	−2.61	0.04	1.22
Ear12	NM_001012766	eosinophil-associated, ribonuclease A family, M 12	−2.29	0.04	1.20
Ehf	NM_007914	ets homologous factor	2.89	0.04	1.16
Ssbp2	NM_024272	single-stranded DNA binding protein 2, TV 2	2.49	0.04	1.14
Phlda3	NM_013750	pleckstrin homology-like domain, family A, M 3	−4.08	0.04	1.14
Whsc1	NM_001177884	Wolf-Hirschhorn syndrome candidate 1, TV 3	2.16	0.04	1.11
Gm2a	NM_010299	GM2 ganglioside activator protein	−2.21	0.04	1.11
Armc2	NM_001034858	armadillo repeat containing 2	3.03	0.04	1.09

Comparison of transcriptoma of c-*myc*-3'RR/Cdk4^R24C^ and c-*myc*-3'RR/p53^+/−^ MCL-like lymphomas. Among these 44,000 genes, 176 significantly differed. A Log score >2, a Log ratio >2 or <−2 and a p<0.05 was used as cut of. A: c-*myc*-3'RR/p53^+/−^ MCL-like lymphomas; B: c-*myc*-3'RR/Cdk4^R24C^ MCL-like lymphomas; M: member; TV: transcript variant.

**Table 2:** Genes implicated in diabetes and obesity

			Log2 ratio	p-value	log score
Name	Systematic Name	Description	A-B	A-B	A-B
Abcc8	NM_011510	ATP-binding cassette, sub-family C, M 8	4.11	0.03	4.02
Ahnak	NM_001039959	nucleoprotein (desmoyokin), TV 3	−4.48	0.03	3.95
Trf	NM_133977	transferrin	−4.41	0.03	3.94
Apoc1	NM_007469	apolipoprotein C-I, TV 1	−3.74	0.03	3.94
Cr2	NM_007758	complement receptor 2	−3.62	0.03	3.77
Cdkn2a	NM_009877	cyclin-dependent kinase inhibitor 2A, TV 1	4.17	0.03	3.45
Gfra2	NM_008115	glial derived neurotrophic factor family receptor alpha 2	−4.48	0.03	3.44
Igf1	NM_010512	insulin-like growth factor 1, TV 1	−3.1	0.03	3.43
Igf2bp3	NM_023670	insulin-like growth factor 2 mRNA binding protein 3	2.85	0.03	3.38
Hmox1	NM_010442	heme oxygenase (decycling) 1	−3.87	0.03	3.29
Gstt1	NM_008185	glutathione S-transferase, theta 1	2.7	0.03	3.17
Rcan2	NM_207649	regulator of calcineurin 2, TV 1	3.87	0.03	3.16
Tub	NM_021885	tubby candidate gene	−3.29	0.03	3.13
Osbpl3	NM_001163645	oxysterol binding protein-like 3, TV 2	2.49	0.03	2.99
Mgll	NM_001166251	monoglyceride lipase, TV 1	2.1	0.03	2.85
Srd5a1	NM_175283	steroid 5 alpha-reductase 1	3.31	0.03	2.42
Rgs16	NM_011267	regulator of G-protein signaling 16	2.96	0.03	2.23
Cbs	NM_144855	cystathionine beta-synthase, TV 1	3.82	0.03	2.19
Alox5	NM_009662	arachidonate 5-lipoxygenase	2.18	0.03	2.19
Pdk1	NM_172665	pyruvate dehydrogenase kinase, isoenzyme 1	2.68	0.03	1.96
Me1	NM_001198933	malic enzyme 1, TV 2	2.49	0.03	1.90
Mef2b	NM_001045484	myocyte enhancer factor 2B, TV 2	3.17	0.03	1.88
Foxp2	ENSMUST00000118133	forkhead box P2 [ENSMUST00000118133]	2.08	0.03	1.79
Aurka	NM_011497	aurora kinase A	2.07	0.03	1.70
Ube2e2	NM_144839	ubiquitin-conjugating enzyme E2E 2	3.03	0.03	1.66
Bmpr1a	NM_009758	bone morphogenetic protein receptor, type 1A	3.25	0.04	1.45
Kcnj10	NM_001039484	potassium inwardly-rectifying channel, subfamily J, M 10	−3.04	0.04	1.39
Hfe	NM_010424	hemochromatosis	−2.07	0.04	1.33
Pdss1	NM_019501	prenyl (solanesyl) diphosphate synthase, subunit 1	2.06	0.04	1.07
Hpgd	NM_008278	hydroxyprostaglandin dehydrogenase 15	−2.94	0.04	1.04
Fabp5	NM_010634	fatty acid binding protein 5	2.65	0.04	1.02

Comparison of transcriptoma of c-*myc*-3'RR/Cdk4^R24C^ and c-*myc*-3'RR/p53^+/−^ MCL-like lymphomas. Among these 44,000 genes, 176 significantly differed. A Log score >2, a Log ratio >2 or <−2 and a p<0.05 was used as cut of. A: c-*myc*-3'RR/p53^+/−^ MCL-like lymphomas; B: c-*myc*-3'RR/Cdk4^R24C^ MCL-like lymphomas; M: member; TV: transcript variant.

**Table 3:** Genes implicated in cellular architecture

			Log2 ratio	p-value	log score
Name	Systematic Name	Description	A-B	A-B	A-B
Ahnak	NM_001039959	nucleoprotein (desmoyokin), TV 3	−4.48	0.03	3.95
Myadm	NM_016969	myeloid-associated differentiation marker, TV 4	−5.5	0.03	3.69
Stab2	NM_138673	stabilin 2	−3.39	0.03	3.50
Fcna	NM_007995	ficolin A	−4.8	0.03	3.46
Avil	NM_009635	advillin	4.05	0.03	3.42
Ctnnbip1	NM_023465	catenin beta interacting protein 1, TV 1	2.32	0.03	2.85
Ncan	NM_007789	neurocan	2.23	0.03	2.78
Kif18b	NM_197959	kinesin family member 18B	2.17	0.03	2.68
Pcolce2	NM_029620	procollagen C-endopeptidase enhancer 2	−2.11	0.03	2.61
Spock2	NM_052994	sparc/osteonectin	2.36	0.03	2.50
Thbs3	NM_013691	thrombospondin 3	−4.07	0.03	2.37
Lmnb1	NM_010721	lamin B1	2.01	0.03	2.30
Zwilch	NM_026507	Zwilch, kinetochore associated	2.25	0.03	2.19
Cd97	NM_011925	CD97 antigen, TV 1	−3.62	0.03	2.07
Mtus2	NM_029920	microtubule associated tumor suppressor candidate 2	−4.03	0.03	1.95
Cenpi	NM_145924	centromere protein I	2.11	0.03	1.94
Mef2b	NM_001045484	myocyte enhancer factor 2B, TV 2	3.17	0.03	1.88
Dscaml1	NM_001081270	down syndrome cell adhesion molecule-like 1	−2.58	0.03	1.81
Tubb2b	NM_023716	tubulin, beta 2B	−4.58	0.03	1.75
Spc25	NM_001199123	NDC80 kinetochore complex component, TV 1	2.23	0.04	1.56
Mtap2	NM_001039934	microtubule-associated protein 2, TV 1	2.24	0.04	1.56
Stmn1	NM_019641	stathmin 1	2.77	0.04	1.39
Slmo1	NM_144867	slowmo homolog 1	4.5	0.04	1.17

Comparison of transcriptoma of c-*myc*-3'RR/Cdk4^R24C^ and c-*myc*-3'RR/p53^+/−^ MCL-like lymphomas. Among these 44,000 genes, 176 significantly differed. A Log score >2, a Log ratio >2 or <-2 and a p<0.05 was used as cut of. A: c-*myc*-3'RR/p53^+/−^ MCL-like lymphomas; B: c-*myc*-3'RR/Cdk4^R24C^ MCL-like lymphomas; M: member; TV: transcript variant.

**Table 4:** Unknown genes

			Log2 ratio	p-value	log score
Name	Systematic Name	Description	A-B	A-B	A-B
Pqlc1	NM_001164420	PQ loop repeat containing 1, TV 2	−3.62	0.03	3.97
NAP101497-1	same	unknown	−2.54	0.03	3.09
LOC100502767	XR_104684	hypothetical LOC100502767	3.01	0.03	3.04
A_55_P1973560	same	unknown	2.29	0.03	2.99
Ng23	NM_023893	Ng23 protein	4.68	0.03	2.92
A_55_P2137023	same	unknown	2.64	0.03	2.87
ENSMUST00000103452	same	predicted gene 16886	−2.55	0.03	2.75
Frmd5	NM_172673	FERM domain containing 5	2.51	0.03	2.56
4931429I11Rik	NM_001081121	RIKEN cDNA 4931429I11 gene	−4.04	0.03	2.46
5730416F02Rik	NR_033596	RIKEN cDNA 5730416F02 gene	−2.66	0.03	2.28
ENSMUST00000098678	same	RIKEN cDNA D930028M14 gene	−2.44	0.03	2.16
ENSMUST00000103381	same	predicted gene 16944	−2.54	0.03	2.15
ENSMUST00000103341	same	predicted gene 16729	−5.03	0.03	2.00
Clip3	NM_001081114	CAP-GLY domain containing linker protein 3	3.96	0.03	1.89
ENSMUST00000103314	same	predicted gene 16798	−4.33	0.03	1.80
ENSMUST00000103348	same	predicted gene 1502	−4.52	0.03	1.76
Cd2	NM_013486	CD2 antigen	−2.97	0.03	1.72
9030409G11Rik	NM_001109685	RIKEN cDNA 9030409G11 gene, TV 3	2.64	0.03	1.69
ENSMUST00000103444	same	predicted gene 16971	−2.46	0.03	1.69
Lrrc23	NM_013588	leucine rich repeat containing 23	−3.82	0.03	1.67
Sssca1	NM_020491	Sjogren's syndrome/scleroderma autoantigen 1 homolog	2.06	0.04	1.64
ENSMUST00000103316	same	predicted gene 5571	−3.83	0.04	1.59
Gm3227	XR_105936	predicted gene 3227	−2.99	0.04	1.46
C77080	NM_001033189	expressed sequence C77080	−3.6	0.04	1.44
2810025M15Rik	NR_027984	RIKEN cDNA 2810025M15 gene	2.05	0.04	1.25
2200002J24Rik	NM_026961	RIKEN cDNA 2200002J24 gene	2.24	0.04	1.25
6030419C18Rik	NM_176921	RIKEN cDNA 6030419C18 gene	2.42	0.04	1.20
ENSMUST00000103493	same	predicted gene 16694	−2.37	0.04	1.16
A_55_P2040519	A_55_P2040519	unknown	2.63	0.04	1.14
A_55_P2121294	same	unknown	−2.46	0.04	1.12
D330028D13Rik	NM_172727	RIKEN cDNA D330028D13 gene, TV 1	2.05	0.04	1.11
LOC100502627	BC058714	cDNA clone IMAGE:6842867	2.26	0.04	1.01

Comparison of transcriptoma of c-*myc*-3'RR/Cdk4^R24C^ and c-*myc*-3'RR/p53^+/−^ MCL-like lymphomas. Among these 44,000 genes, 176 significantly differed. A Log score >2, a Log ratio >2 or <-2 and a p<0.05 was used as cut of. A: c-*myc*-3'RR/p53^+/−^ MCL-like lymphomas; B: c-*myc*-3'RR/Cdk4^R24C^ MCL-like lymphomas; M: member; TV: transcript variant.

Differences concerning genes involved in diabetes and obesity (such as Abcc8, Trf, apoc1 and IGF-1) appear directly linked to the Cdk4^R24C^ mutation since loss of Cdk4 expression causes insulin-deficient diabetes and Cdk4 activation results in β-islet cell hyperplasia [10]. The metabolic and endocrinic changes resulting from diabetes and obesity may dysregulate DNA repair, gene functions and cell mutation rate favouring neoplastic transformation and leading to hematologic malignancy and cancer [11]. For example IGF-1 (insulin growth factor 1) transcripts are markedly elevated in MCL-like lymphomas of c-*myc*-3'RR/Cdk4^R24C^ mice; IGF-1 involvement being well documented in cancer [11]. Differences concerning genes implicated in the growth and signalling processes could be explained by the Cdk4^R24C^ and p53 mutations themselves. For example, up-regulation of the cell cycle regulatory genes Ccnb1 and Cdc20 in MCL-like lymphomas of c-*myc*-3'RR/p53^+/−^ mice appear directly linked to the p53^+/−^ mutation that increases the rate and occurrence of c-*myc*-induced lymphomas [6, 12]. Indeed, Ccnb1 overexpression in lymphomas is caused by non-functional p53 [13], while Cdc20 is negatively regulated by p53 [14]. In turn the down regulation of the Cdk4 inhibitor Cdkn2a in MCL-like lymphomas of c-*myc*-3'RR/Cdk4^R24C^ mice appears related to its inefficiency in Cdk4^R24C^ mice. As a consequence of a higher proliferation rate, several genes implicated in cell metabolism (such as Adarb1, Lmo1, AK4, Slc2a4) and nuclear membrane or chromosome stability (such as Lmnb1 and Cenpi) are higher in MCL-like lymphomas of c-*myc*-3'RR/p53^+/−^ mice than in MCL-like lymphomas of c-*myc*-3'RR/Cdk4^R24C^ mice. Finally and also linked to a higher rate of proliferation, several differences are found concerning genes implicated in cellular architecture especially on the actin and microtubule cytoskeletons (such as advillin Kif18b, Mtus2, Tubb2b, Mtap2, Stmn1), key players that underpin growth processes [15].

MCL-like lymphomas of c-*myc*-3'RR/p53^+/−^ mice are more aggressive than those of c-*myc*-3'RR/Cdk4^R24C^ mice despite similar flow cytometry profiles [5, 6]. Comparison of their gene expression profiles explains this difference by a marked overexpression of several cell cycle regulatory genes. Gene expression profiles do not highlight other specific differences other than those in relation with their specific mutational status (*i.e.,* Cdk4^R24C^ or p53 mutations). We propose that similarly to typical human MCL and its blastoid or cyclin-D1 variants that correspond to the same genetic entity [1], MCL-like lymphomas of c-*myc*-3'RR/p53^+/−^ mice and c-*myc*-3'RR/Cdk4^R24C^ mice represent a spectrum of the same entity. Our results indicate that deregulation of two different signalling pathways within a single B cell entity can lead to the emergence of a unique lymphoma phenotype carrying different oncogenic stigmas. These different oncogenic stigmas explain differences concerning the proliferative and/or apoptotic status of the lymphoma and thus potential differential responses to treatment. Tumor transcriptoma analysis and tumor DNA sequence analysis could thus become useful laboratory tests paving the way towards personalized treatments [16].
